# The inhibition of pancreatic cancer progression by K-Ras-overexpressing mesenchymal stem cell-derived secretomes

**DOI:** 10.1038/s41598-023-41835-6

**Published:** 2023-09-12

**Authors:** Qingji Huo, Kexin Li, Xun Sun, Adam Zhuang, Kazumasa Minami, Keisuke Tamari, Kazuhiko Ogawa, Melissa L. Fishel, Bai-Yan Li, Hiroki Yokota

**Affiliations:** 1 Department of Pharmacology, School of Pharmacy, Harbin Medical University, Harbin, 150081 China; 2 Department of Biomedical Engineering, Indiana University Purdue University Indianapolis, Indianapolis, IN 46202 USA; 3 Department of Radiation Oncology, Osaka University Graduate School of Medicine, Suita, Osaka 565-0871 Japan; 4 Department of Pediatrics, Wells Center for Pediatric Research, Indiana University School of Medicine, Indianapolis, IN 46202 USA; 5 Department of Pharmacology and Toxicology, Indiana University School of Medicine, Indianapolis, IN 46202 USA; 6 Indiana University Simon Comprehensive Cancer Center, Indianapolis, IN 46202 USA; 7 Indiana Center for Musculoskeletal Health, Indiana University School of Medicine, Indianapolis, IN 46202 USA

**Keywords:** Diseases, Oncology

## Abstract

Pancreatic ductal adenocarcinoma (PDAC) is an aggressive cancer with poor survival. To explore an uncharted function of K-Ras proto-oncogene, K-Ras was activated in mesenchymal stem cells (MSCs) and the effects of MSC conditioned medium (CM) on PDAC were examined. Overexpression of K-Ras elevated PI3K signaling in MSCs, and K-Ras/PI3K-activated MSC-derived CM reduced the proliferation and migration of tumor cells, as well as the growth of ex vivo freshly isolated human PDAC cultures. CM’s anti-tumor capability was additive with Gemcitabine, a commonly used chemotherapeutic drug in the treatment of PDAC. The systemic administration of CM in a mouse model suppressed the colonization of PDAC in the lung. MSC CM was enriched with Moesin (MSN), which acted as an extracellular tumor-suppressing protein by interacting with CD44. Tumor-suppressive CM was also generated by PKA-activated peripheral blood mononuclear cells. Collectively, this study demonstrated that MSC CM can be engineered to act as a tumor-suppressive agent by activating K-Ras and PI3K, and the MSN-CD44 regulatory axis is in part responsible for this potential unconventional option in the treatment of PDAC.

## Introduction

Pancreatic ductal adenocarcinoma (PDAC) is the third leading cause of cancer death in the United States with a five-year survival rate of around 11%^1–3^. It provides few early signs and spreads rapidly to surrounding organs even after surgical resection. The recurrence sites after resection include the liver, lung, bone, peritoneum, and multiple other organs^4^. Currently, surgery along with adjuvant and neo-adjuvant chemotherapies is the primary treatment option for patients with non-metastatic pancreatic cancer but surgical resection needs to be performed early and is not always a viable option due to early systemic dissemination of the disease^5–7^. Metastatic or recurrent pancreatic cancer can be treated via first-line chemotherapy agents such as FOLFIRINOX, gemcitabine plus nab-paclitaxel, or erlotinib^8–11^. While these treatments have marginally improved outcomes for pancreatic cancer patients, the long-term survival rate is still very low, indicating that newer, more effective systemic therapies and treatment strategies are required.

For advanced and metastatic breast cancer, prostate cancer as well as osteosarcoma, an unconventional treatment strategy using “induced tumor-suppressing cells” (iTSCs) has been proposed^12^. iTSCs can be generated from various cell types, including mesenchymal stem cells (MSCs), osteocytes, osteoblasts, and peripheral blood mononuclear cells, as well as breast and prostate cancer cells^13–18^. It requires the activation of cell proliferating and oncogenic pathways, for instance, by overexpressing β-catenin in Wnt signaling, and Snail in the epithelial-to-mesenchymal transition^13^. Our previous studies have suggested that iTSCs could be generated by transfecting MSCs with Lrp5, β-catenin, Snail, or Akt^12,14,15,19,20^. The gene overexpression approach and a systemic administration of MSC CMs effectively inhibited the growth of mammary tumors and tumor-induced osteolysis in mouse models^19^. Here, we examined the conversion of MSCs and peripheral blood mononuclear cells (PBMCs) into iTSCs for the potential treatment of PDAC. Autologous MSCs can be isolated from the bone marrow and adipose tissue of cancer patients, while autologous PBMCs can be harvested from the peripheral blood of patients with pancreatic cancer. MSC-derived CM has been previously used as a therapeutic option in other pathologies. For example, growth factors in MSC-derived CM are effective in bone regeneration^21^, and the application of CM from adipose-derived stem cells can effectively enhance wound healing^22^. To the best of our knowledge, however, the application of MSC/PBMC-derived CM for the treatment of pancreatic cancer has not been explored.

In about 90% of PDAC cases, a proto-oncogene K-Ras is mutated^23,24^. While it occurs only in a few percentages of pancreatic cancer^25^, a mutation in K-Ras G12C accounts for about 44% of all K-Ras mutations^26^. Its inhibitor, Sotorasib, was recently tested for K-Ras G12C-mutated advanced pancreatic cancer^25^. As a GTPase and downstream effector of epidermal growth factor (EGF) signaling, K-Ras gain-of-function mutations constitutively activate intracellular,tumorigenic signaling cascades, with some of the most prominent being controlled by phosphatidylinositol 3 kinase (PI3K)^27,28^. PI3K is important in cell cycle checkpoints and in preventing apoptosis in cancer cells^29^. There was significant research on selective K-Ras inhibitors with a breakthrough occurring in 2013 with the discovery of the K-Ras switch II pocket^30^. In 2021, Sotorasib became the first FDA-approved drug to directly target tumors with K-Ras mutations. The same year, Adagrasib received therapy designation from the FDA^30^. However, selective K-Ras inhibitors as with other chemotherapy agents lead to adaptive resistance, and therefore research is being done on newer K-Ras inhibitors and different combination therapies^30,31^.

In contrast to directly inhibiting K-Ras, our study sought to activate K-Ras-linked pathways due to previous findings that the overexpression of tumor-promoting transcription factors such as Oct4 and cMyc in tumor cells and MSCs can generate anti-tumor CM^17^. Notably, the effect of the overexpressed cMyc was first reported in Drosophila. When a group of Drosophila cells expressed a higher level of d-myc (a homolog of cMyc), they secreted a group of proteins and eliminated neighboring wild-type cells^32,33^. Applying the same idea to treating pancreatic cancer, the present study examined the novel approach of activating K-Ras signaling in MSCs and PBMCs, generating CM, and analyzing the downstream effects of MSC CM on pancreatic cancer cell growth and migration. K-Ras is known to deregulate p-Akt in PI3K signaling, as well as p-CREB in cAMP/protein kinase A (PKA) signaling^34,35^. Here, we generated tumor-suppressive CM from MSCs treated with YS49 as a pharmacological PI3K activator, and from PBMCs treated with CW008 as a selective PKA activator.

In our previous studies, global proteomics analysis revealed that iTSC CM was enriched with atypical tumor-suppressing proteins such as Hsp90ab1, Enolase 1, Ubiquitin C, and Moesin (MSN)^15–18,36^. Here, we focused on the anti-tumor effect of extracellular MSN. Since CD44 is a cell surface adhesion receptor, we evaluated the potential role of the MSN-CD44 regulatory axis in the observed tumor-suppressing capabilities and expression of K-Ras in PDAC cells. Of note, MSN is reported to interact with CD44^15^. Taken together, this study demonstrated the anti-tumor action of K-Ras, PI3K-activated MSC CM, and PKA-activated PBMC CM, indicating the possibility of unconventional proteome-based therapy for pancreatic cancer.

## Results

### Double-edged role of K-Ras

**Figure 1:**
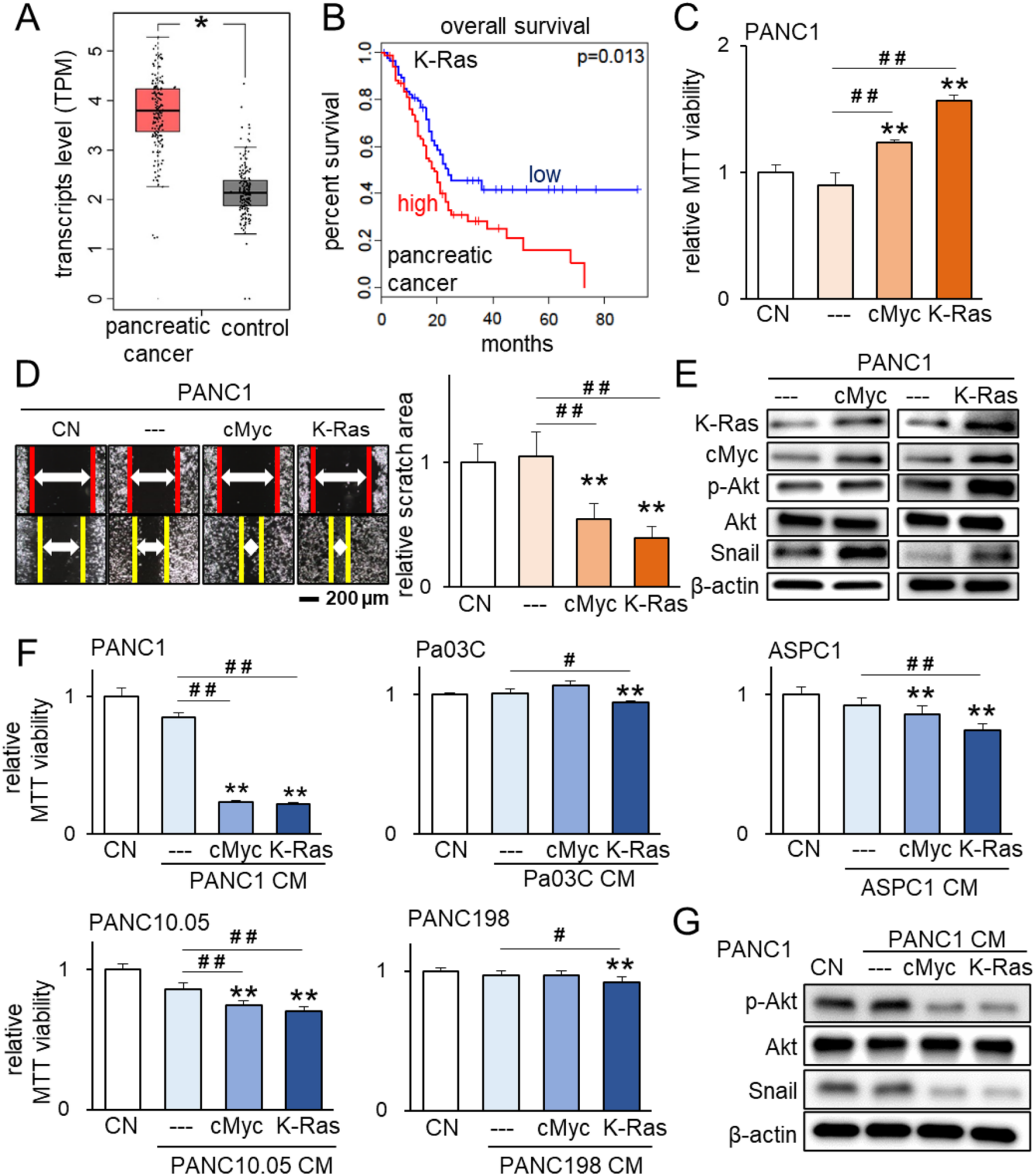
Tumor-suppressing capability of K-Ras and cMyc-overexpressing cell-derived CM. The single and double asterisks (comparison to CN) and pound signs (comparison to CM control) indicate p < 0.05 and 0.01, respectively. *CM* conditioned medium, *CN* control. (**A**) Elevation of K-Ras transcripts in pancreatic cancer tissues in the TCGA database. (**B**) Poor survival rate for pancreatic cancer patients with a high level of K-Ras (p = 0.013). (**C**,**D**) Increase in the MTT-based viability and 2-dimensional motility of PANC1 pancreatic cancer cells by the overexpression of cMyc and K-Ras. (**E**) Elevated levels of K-Ras, cMyc, p-Akt, and Snail by the overexpression of cMyc and K-Ras in PANC1 cells. (**F**) Suppression of MTT-based viability of 5 pancreatic cancer cell lines (PANC1, Pa03C, ASPC1, PANC10.05, and PANC198) by CM derived from cMyc or K-Ras-overexpressing cancer cells. Of note, cMyc overexpression was not effective in Pa03C and PANC198 cells, but K-Ras overexpression was effective in all cell lines. (**G**) Reduction in p-Akt and Snail in PANC1 cells by cMyc- or K-Ras-overexpressing PANC1-derived CM.

According to the TCGA database, the transcript level of K-Ras proto-oncogene is elevated in pancreatic cancer tissues, and its elevation is linked to the poor survival rate of patients with pancreatic cancer (p = 0.013; Fig. 1A,B). Consistently, the overexpression of K-Ras, as well as cMyc, another proto-oncogene, in PANC1 pancreatic cancer cells promoted MTT-based cellular viability and scratch-based cellular motility (Fig. 1C,D). Furthermore, their overexpression upregulated the phosphorylated form of Akt (p-Akt) in PI3K signaling as well as Snail, which is linked to EMT (Fig. 1E). By contrast, the effect of the conditioned medium (CM), which was harvested from K-Ras and cMyc overexpressing pancreatic cancer cells, was sharply opposite. K-Ras-overexpressing PANC1-derived CM suppressed MTT-based viability of cohort PANC1 cells, and the same effect was observed by cMyc-overexpressing PANC1-derived CM (Fig. 1F). The reduction of EdU-based proliferation of PANC1 cells was also observed by the application of cMyc- and K-Ras-overexpressing PANC1-derived CM (Supplementary Fig. 1). The tumor-suppressive effect of K-Ras overexpression was also shown in 4 other pancreatic cancer cell lines, including Pa03C, ASPC1, PANC10.05, and PANC198, as well as MDA-MB-231 breast cancer cells (Fig. 1F; Supplementary Fig. 2). The levels of p-Akt and Snail were downregulated in PANC1 cells by the application of cMyc- and K-Ras-overexpressing PANC1-derived CM (Fig. 1G). The same reduction was observed in MDA-MB-231 cells by their cMyc- and K-Ras-overexpressing CM (Supplementary Fig. 2D). Collectively, the results presented the striking difference in the role of K-Ras overexpression in pancreatic cancer cells and their CM.

### Tumor-suppressing CM by PI3K-activated MSCs

**Figure 2:**
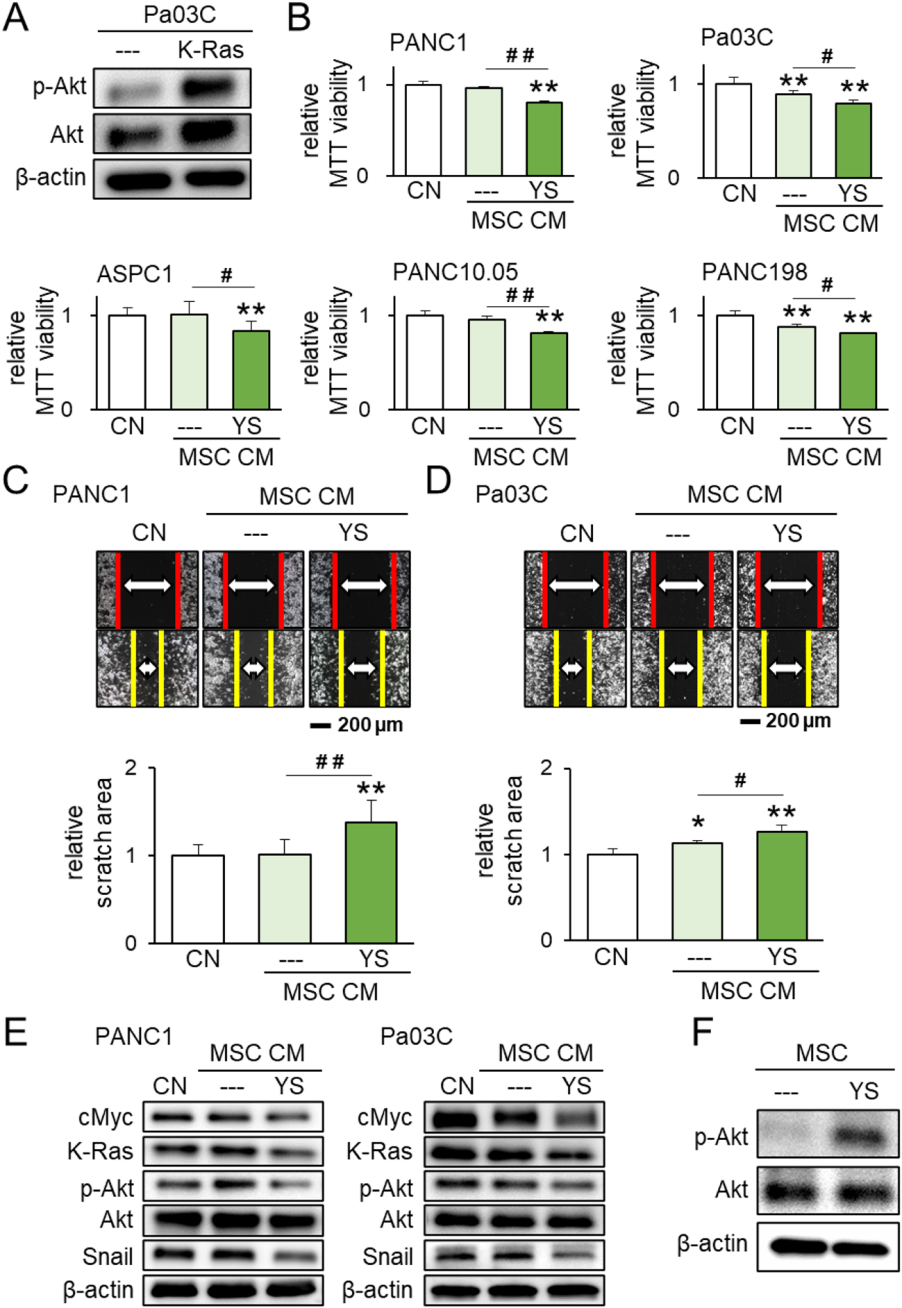
Tumor-suppressing capability of PI3K-activated MSC-derived CM. The single and double asterisks (comparison to CN) and pound signs (comparison to CM control) indicate p < 0.05 and 0.01, respectively. *CM* conditioned medium, *CN* control, *YS* YS49, PI3K activator. (**A**) Elevation of p-Akt by K-Ras-overexpressing Pa03C pancreatic cancer cells. (**B**) Suppression of MTT-based viability of PANC1, Pa03C, ASPC1, PANC10.05, and PANC198 pancreatic cancer cells by YS49-treated MSC-derived CM. (**C**,**D**) Suppression of scratch-based migration in PANC1 cells and Pa03C cells, respectively, by YS49-treated MSC-derived CM. (**E**) Reduction in cMyc, K-Ras, p-Akt, and Snail in PANC1 and Pa03C pancreatic cancer cells by YS49-treated MSC-derived CM. (**F**) Elevation of p-Akt in YS49-treated MSCs.

We next examined the effect of PI3K activation on tumor progression since the overexpression of K-Ras elevated p-Akt in PI3K signaling (Fig. 2A). Of note, the level of p-Akt was also elevated by the overexpression of cMyc and K-Ras in ASPC1, PANC10.05, and PANC198 cell lines (Supplementary Fig. 3A). As CM-producing cells, we selected bone marrow-derived MSCs because of their current usage for regenerative medicine^37^ and potential usage for cancer treatments. The treatment of MSCs with 50 μM of YS49, a pharmacological PI3K activator, generated the tumor-suppressing CM, and the application of YS49-treated MSC-derived CM reduced the MTT-based viability of 5 pancreatic cancer cell lines (Fig. 2B). Furthermore, YS49-treated MSC-derived CM decreased the scratch-based motility of PANC1 and Pa03C cells (Fig. 2C,D), as well as EdU-based proliferation of PANC1 cells (Supplementary Fig. 3B). Additionally, PI3K-activated MSC CM downregulated the expression of cMyc, K-Ras, p-Akt, and Snail in PANC1 and Pa03C cells (Fig. 2E). By contrast, the level of p-Akt was elevated in YS49-treated MSCs (Fig. 2F).

### Inhibition of tumor invasion by PI3K-activated MSC CM

**Figure 3:**
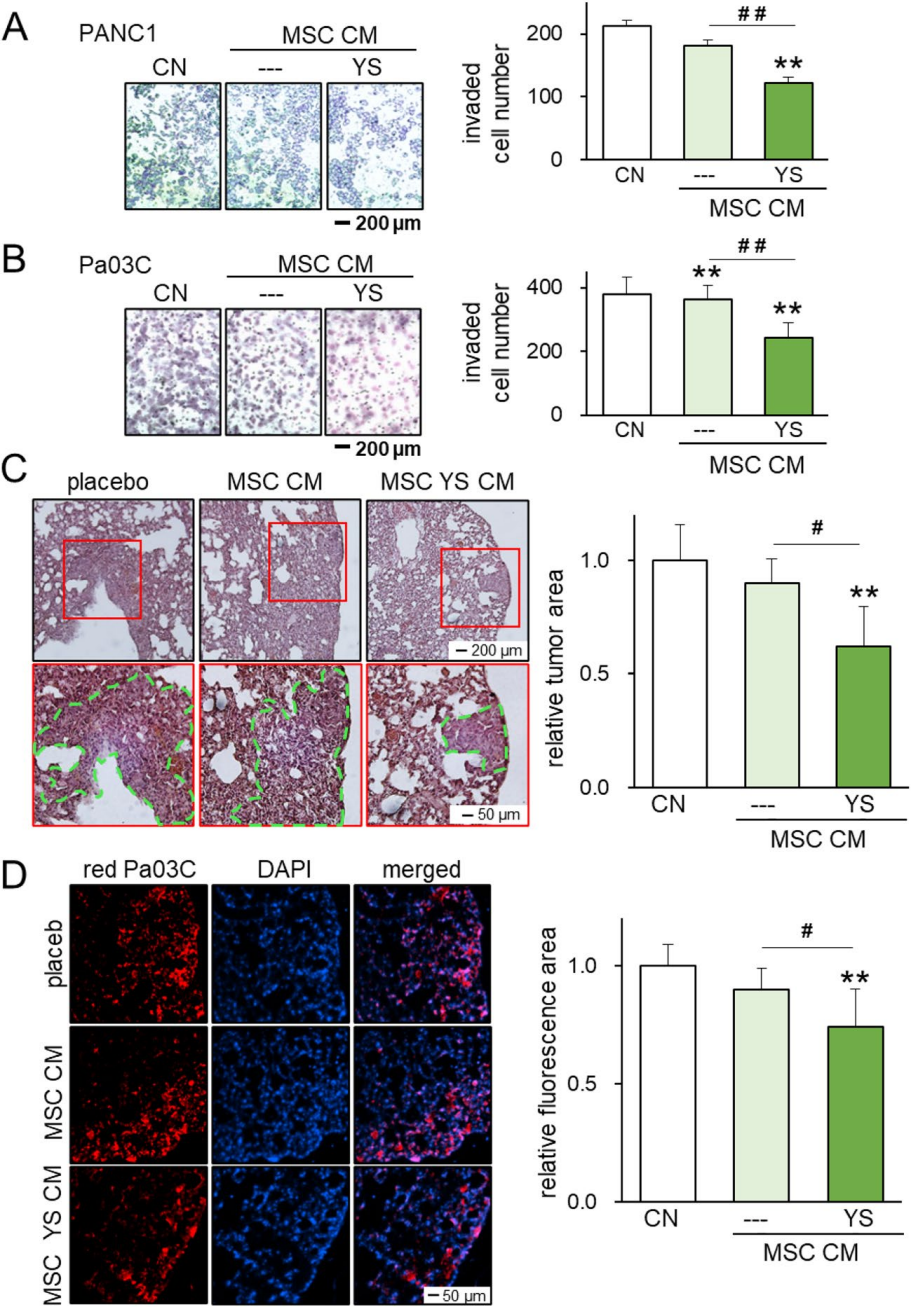
Suppression of Pa03C tumor cell growth by PI3K-activated MSC-derived CM. The single and double asterisks (comparison to CN) and pound signs (comparison to CM control) indicate p < 0.05 and 0.01, respectively. *CM* conditioned medium, *CN* control, *YS* YS49, PI3K activator. (**A**,**B**) Suppression of transwell invasion of PANC1 and Pa03C cells, respectively, by PI3K-activated MSC-derived CM. (**C**) HE-stained lung sections of NOD/SCID/γ(−/−) mice, showing the suppression of cell growth by YS49-treated MSC CM. Pa03C cells were inoculated from the tail vein (N = 5). (**D**) Fluorescently labeled lung sections of three mouse groups. Pa03C cells were labeled with red fluorescent proteins and all nuclei were stained with DAPI.

In addition to reducing cell viability and motility, PI3K-activated MSC CM also suppressed transwell invasion of PANC1 and Pa03C cells (Fig. 3A,B). Furthermore, we employed an NSG mouse model, and fluorescently labeled Pa03C pancreatic cancer cells were inoculated via tail-vein injection. After treatment with a placebo, MSC CM, or CM generated from MSCs treated with YS49, we observed a significant reduction in the number of metastatic lung lesions. Histological analysis with H&E-stained lung sections supported the anti-tumor effect of YS49-treated MSC-derived CM, which significantly reduced the tumor-invaded area (p < 0.01) (Fig. 3C,D).

### Anti-tumor role of the MSN-CD44 regulatory axis

**Figure 4:**
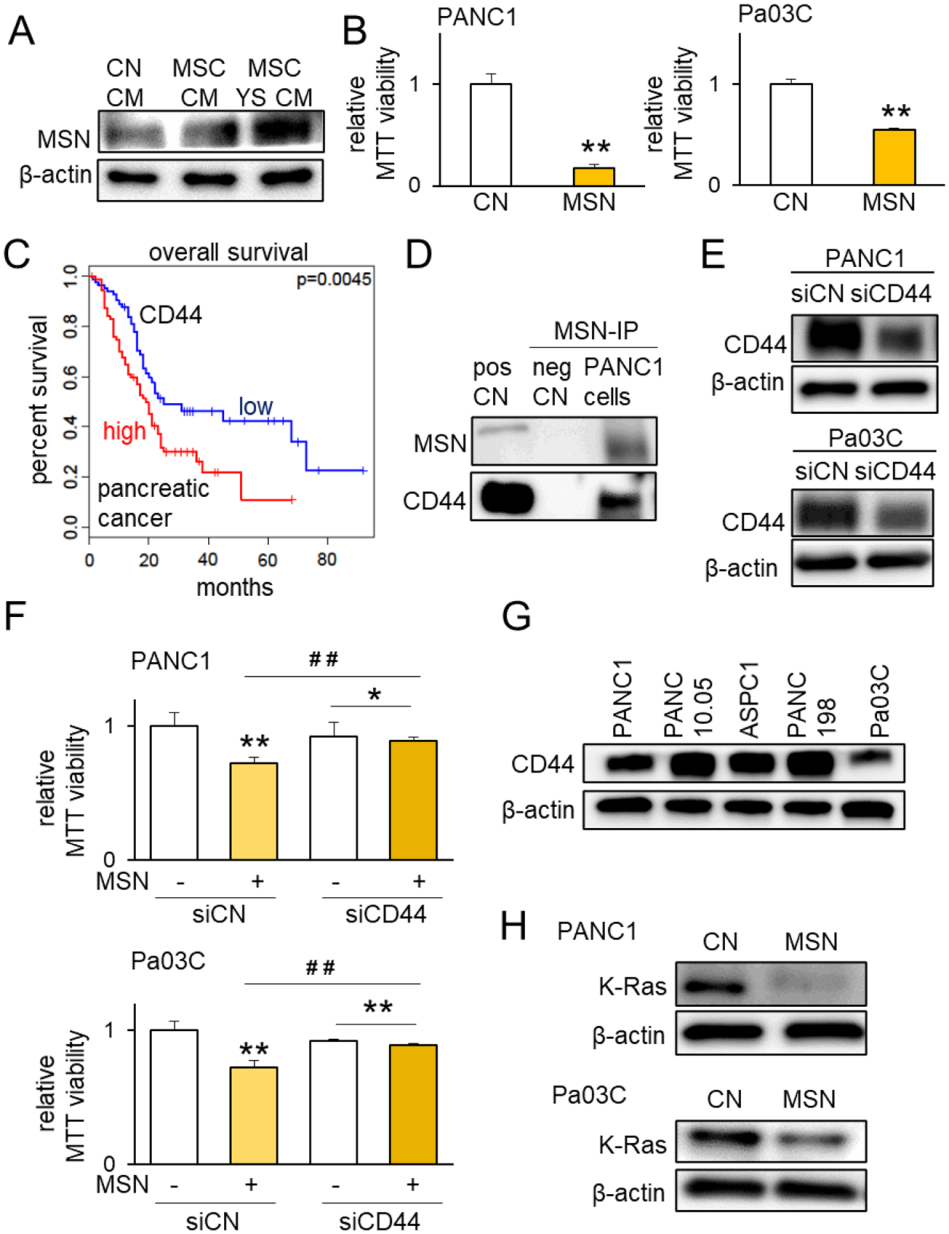
Role of the Moesin (MSN)-CD44 regulatory axis. The single and double asterisks (comparison to CN) and pound signs (comparison to siRNA control) indicate p < 0.05 and 0.01, respectively. *CM* conditioned medium, *CN* control, *YS* YS49, PI3K activator, and si = siRNA. (**A**) Elevated level of MSN in YS49-treated MSC-derived CM. (**B**) Reduction in the MTT-based viability of PANC1 and Pa03C pancreatic cancer cells in response to 1 µg/mL of recombinant MSN proteins. (**C**) Low survival rate of pancreatic cancer patients with high levels of MSN (p = 0.0045). (**D**) Immunoprecipitation of CD44 by MSN in PANC1 cell lysates. (**E**,**F**) Suppression of MSN-driven reduction in the MTT-based viability of PANC1 and Pa03C pancreatic cancer cells by silencing CD44. (**G**) Levels of CD44 in the five pancreatic cell lines. (**H**) Reduction of K-Ras in PANC1 and Pa03C cells in response to 1 µg/mL of recombinant MSN proteins.

Using mass spectrometry-based proteomics analysis, we previously identified MSN as one of the tumor-suppressing proteins that were enriched in the tumor-suppressive CM^38^. In YS49-treated MSC CM, MSN was elevated (Fig. 4A). The elevation of MSN was also observed in cMyc- and K-ras-overexpressing PANC1-derived CM (Supplementary Fig. 4A). And 1 µg/mL of MSN significantly reduced the MTT-based viability of PANC1 and Pa03C cells (Fig. 4B), as well as ASPC1 and PANC198 cells (Supplementary Fig. 4B). CD44 is a cell-surface glycoprotein involved in cell–cell interactions, and its high transcript level in pancreatic cancer tissues, which is linked to a poor survival rate for patients with pancreatic cancer, was shown in the TCGA database (Fig. 4C). The co-immunoprecipitation of CD44 with MSN using PANC1 protein extracts supported the predicted interaction of MSN with CD44 (Fig. 4D). Consistently, silencing of CD44 by siRNA suppressed MSN-driven reduction in MTT-based viability of PANC1 and Pa03C pancreatic cancer cells (Fig. 4E–G). Notably, the treatment of these two pancreatic cancer cell lines with extracellular MSN downregulated K-Ras, indicating the outcome of inhibiting K-Ras for preventing the progression of pancreatic cancer cells (Fig. 4H).

### Additive effect of MSC CM and MSN with gemcitabine

**Figure 5:**
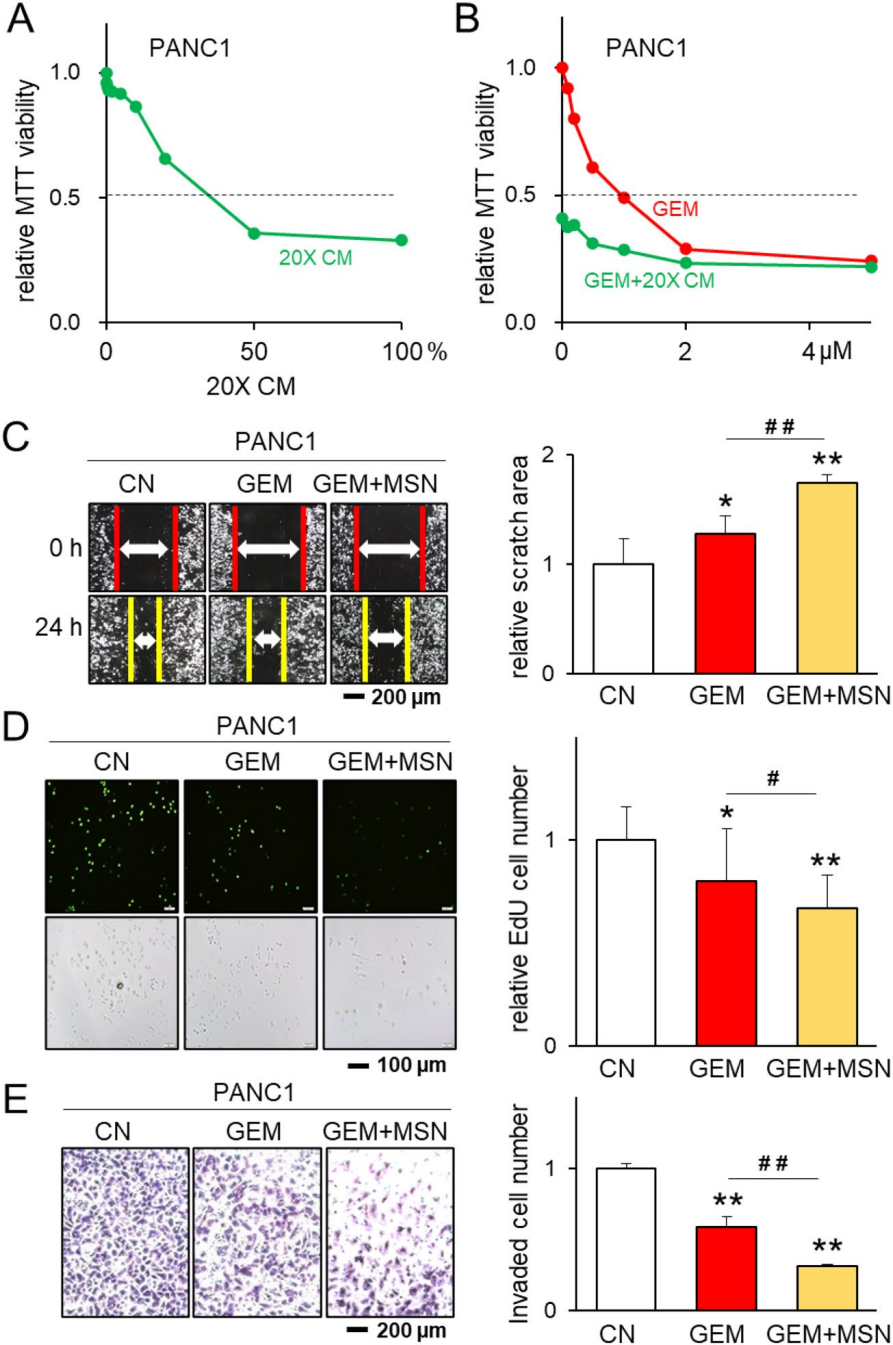
Additive anti-tumor effects of YS49-treated MSC CM and recombinant MSN proteins with Gemcitabine. The single and double asterisks (comparison to CN) and pound signs (comparison to GEM) indicate p < 0.05 and 0.01, respectively. *CM* conditioned medium, *CN* control, *GEM* Gemcitabine. (**A**) MTT-based viability of PANC1 pancreatic cancer cells in response to the condensed CM up to 20-fold. (**B**) Combined effects of 0–5 µM Gemcitabine with 20-fold condensed CM. (**C**–**E**) Additive anti-tumor effects of 0.5 µM of Gemcitabine and 1 µg/mL of recombinant MSN proteins in the scratch-based motility, EdU-based proliferation, and transwell invasion of PANC1 pancreatic cancer cells, respectively.

**Figure 6:**
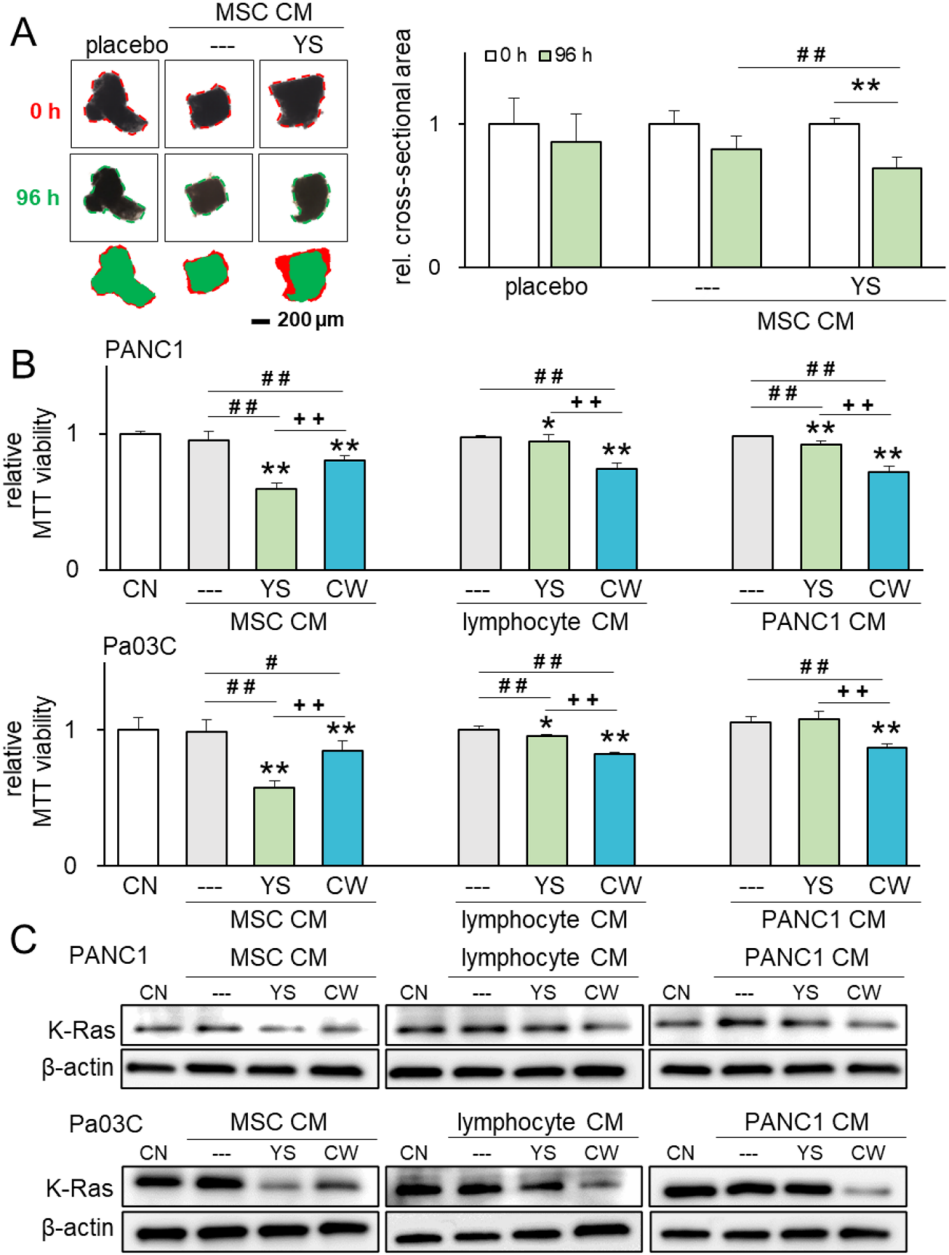
Efficacy in the freshly isolated pancreatic cancer tissue fragments and the generation of the diverse tumor-suppressive CMs. The single and double asterisks (comparison to CN), pound signs (comparison to CM control) and plus signs (comparison to YS) indicate p < 0.05 and 0.01, respectively. *CM* conditioned medium, *CN* control, *YS* YS49, PI3K activator, and *CW* CW008, PKA activator. (**A**) Diminished pancreatic cancer tissue fragments by the application of YS49-treated MSC CM in 96 h (N = 8). The red image indicates tissue fragments at 0 h, while the green image indicates tissue fragments at 96 h. (**B**) Generation of diverse tumor-suppressive CM from MSCs, Jurkat T lymphocytes, and PANC1 pancreatic cancer cells by activating PI3K signaling with 50 µM of YS49 and PKA signaling with 20 µM of CW008. The anti-tumor effects of varying CM were evaluated by the MTT-based viability assay with PANC1 and Pa03C pancreatic cancer cells. (**C**) Reduction in the level of K-Ras in PANC1 and Pa03C pancreatic cancer cells in response to the diverse tumor-suppressive CM.

The tumor-suppressive capability of MSC CM was dose-dependent, and the MTT-based IC_50_ for PANC1 cells was 7.0 × CM (Fig. 5A), indicating that sevenfold condensed CM inhibited 50% of cell viability. When 20 × CM was applied with Gemcitabine, the efficacy was additive and most eminent at 2 µM of Gemcitabine or lower (Fig. 5B). The concentration was selected based on the MTT-based viability assay. The add-on effect was also observed by the simultaneous application of 1 µg/mL of MSN with 0.5 µM of Gemcitabine in the scratch-based motility, EdU-based proliferation, and transwell invasion assays with PANC1 pancreatic cancer cells (Fig. 5C–E). Importantly, the ex vivo fragments of freshly isolated pancreatic cancer tissue were diminished by YS49-treated MSC CM in 4 days (Fig. 6A).

### Generation of iTSCs by activating PKA signaling in MSCs, lymphocytes, and PANC1 cells

While iTSCs and tumor-suppressive CM were generated from MSCs, Jurkat T lymphocytes, and PANC1 cancer cells, the anti-tumor efficacy of CM differed depending on the cell type that the CM was generated. Judging from the MTT-based viability of PANC1 and Pa03C pancreatic cancer cells, PI3K-activated MSC CM with 50 µM of YS49 was more effective than PKA-activated CM with 20 µM of CW008 (Fig. 6B). By contrast, CW008-treated lymphocyte- and PANC1-derived CM showed a stronger anti-tumor effect than their YS49-treated counterparts (Fig. 6B). Consistently, the most effective CM correlated with a reduction in the level of K-Ras in PANC1 and Pa03C cells (Fig. 6C).

### Generation of iTSCs from MSCs, lymphocytes, and PBMCs

**Figure 7:**
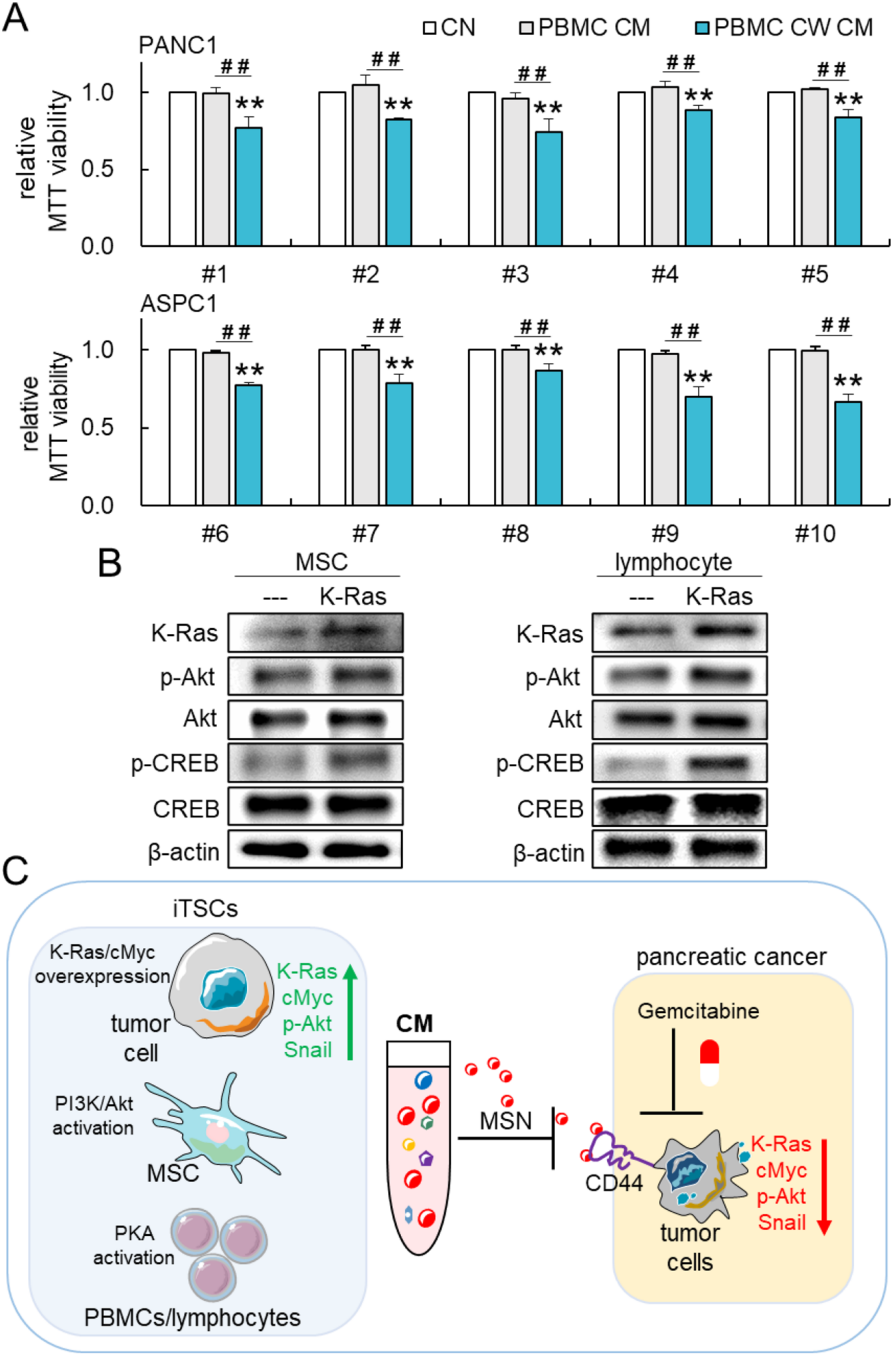
Generation of tumor-suppressive CM from peripheral blood mononuclear cells (PBMCs). The single and double asterisks (comparison to CN) and pound signs (comparison to PBMC CM) indicate p < 0.05 and 0.01, respectively. *CM* conditioned medium, *CN* control, *PBMC* peripheral blood mononuclear cells, *CW* CW008, PKA activator. (**A**) Reduction of the MTT-based viability of PANC1 and ASPC1 pancreatic cancer cells by CW008-treated PBMC-derived CM. (**B**) Elevated levels of K-Ras, p-Akt, and p-CREB by the overexpression of K-Ras in MSCs and Jurkat T-lymphocytes. (**C**) Proposed regulatory mechanism of iTSC CM. The MSN-CD44 regulatory axis is highlighted since MSN is one of the tumor-suppressing proteins that are enriched in the described tumor-suppressive CM.

Thus far, iTSCs and their anti-tumor CM were produced using various non-tumor and tumor cell lines. We also generated anti-PDAC CM from PBMCs that were harvested from 10 healthy individuals. Isolated PBMCs were treated with 50 µM of CW008 for a day. PBMCs were rinsed to remove CW008, and they were cultured in a fresh medium for a day to generate tumor-suppressive CM. The results showed that each of the five PBMC-derived CMs was capable of reducing the MTT-based viability of PANC1 and ASPC1 cell lines, respectively (Fig. 7A).

Lastly, we examined the linkage of K-Ras to PI3K/Akt and PKA/CREB signaling pathways. In MSCs and Jurkat T-lymphocytes, the overexpression of K-Ras elevated the level of p-Akt and p-CREB (Fig. 7B). The results indicate the tumorigenic network of K-Ras, PI3K/Akt, and PKA/CREB in MSCs and lymphocytes.

## Discussion

This study demonstrated the anti-tumor capability of the engineered CM, which was produced from MSCs and PBMCs. Tumor-suppressive CM was enriched with MSN, whose anti-tumor action was mediated by CD44, a cell-surface adhesion receptor. It is reported that PDAC cells expressing a high level of CD44 are highly invasive and they develop Gemcitabine resistance^39^. The engineered MSC-derived CM suppressed the invasion of Pa03C cells in the lung in the mouse model, and it significantly diminished the growth of ex vivo cultures of freshly isolated PDAC tissue. Furthermore, engineered CM showed an additive anti-tumor effect when used simultaneously with Gemcitabine. Besides MSC-derived CM, PBMCs from healthy individuals were converted to iTSCs and their CM also presented potent anti-PDAC capabilities. Collectively, the described iTSC-based strategy presents a unique therapeutic possibility for pancreatic cancer (Fig. 7C). The activation of multiple pathways can generate iTSCs, but the capability of tumor-suppressing CM differs depending on the choice of host iTSCs and a pathway to be activated. In this study, we mainly focused on K-Ras, PI3K, and PKA for PDAC cells, MSCs, and PBMCs, respectively.

Despite a rare G12C mutation in pancreatic cancer, K-Ras is mutated in over 90% of pancreatic cancer cells, and a major effort has been targeted to inhibit its oncogenic signaling. This study took a counterintuitive approach and utilized its aggressive capability of eliminating less-fit cancer cells. Furthermore, this study revealed that the overexpression of K-Ras in MSCs and lymphocytes activated PI3K/Akt and PKA/CREB signaling pathways, and their activation by pharmacological agents also generated tumor-suppressive CM. The results herein are consistent with our previous studies for breast cancer, in which the activation of PI3K converted MSCs into iTSCs, and the progression of breast cancer and bone metastasis was inhibited^16,19^.

The elevated level of MSN in PI3K-activated MSC-derived CM was consistent with its tumor-suppressive actions on PANC1 and Pa03C pancreatic cancer cells. Immunoprecipitation supported MSN’s interaction with CD44, a non-kinase transmembrane glycoprotein^40^. CD44 is overexpressed in many types of cancer including pancreatic cancer^41^, and its high expression level in pancreatic cancer led to poor survival (p = 0.0045). Consistent with the anti-tumor role of MSN, the application of recombinant MSN proteins downregulated K-Ras in PANC1 and Pa03C pancreatic cancer cells.

Currently, research is being done on the use of FDA-approved K-Ras inhibitors and new immunotherapy techniques to try and selectively treat pancreatic cancer^25,42–44^. Sotorasib, which has already been approved to treat small-cell lung cancer, is being examined for its efficacy in treating pancreatic cancer^25,45^. However, trials have only been shown to limit disease progression slightly in a small sample population with stage IV PDAC. Regarding new immunotherapy techniques, studies look at targeting specific antigens on MHCII receptors on tumor cells and also using TCR gene therapy to give immune cells specific TCRs capable of recognizing PDAC cells^43,44^. However, a lot of immunotherapy options are limited by the specific HLA alleles an individual has, and therefore T cell-based immunotherapy needs to be personalized for each individual to work^43,44^.

While immunotherapy is a promising avenue for the future of pancreatic cancer regarding selectivity, we predict that the use of conditioned media for the treatment of pancreatic cancer can be selective with low side effects, but can also be used on any individual due to the use of their own autologous MSCs. Of note, in our previous study, we showed that iTSC CM can act as an inhibitor of programmed cell death ligand 1 (PD-L1) in breast cancer^17–19^. Using conditioned media to treat diseases is not a novel idea. In Indonesia, there are currently clinical trials ongoing to determine the therapeutic potential of stem cell-derived CM on ulcers, cerebral palsy, and even osteoarthritis (NCT04314661, NCT04314687, NCT04134676). Alternatively, as a protein-based therapeutic option, the selected group of tumor-suppressing proteins that are enriched in the described CM can be delivered as a protein cocktail. Further studies are recommended for the development of protein cocktails that retain anti-tumor capabilities without inducing unwanted immune responses.

This study is not free from limitations. First, we employed five PDAC cell lines in cell culture and animal studies without using primary cells. Second, in multiple tumor-suppressive CM derived from PDAC cells, MSCs, lymphocytes, and PBMCs, we focused on the anti-tumor action of the MSN-CD44 regulatory axis. Based on the mixture of varying proteins in iTSC CMs, many other tumor-suppressing proteins are enriched in CM which also likely contribute to the tumor-suppressive CM. Proteomes were studied in this study since the removal of exosomes, nucleic acids, and small biomolecules smaller than 3kD, including nutrient molecules such as glucose an galactose, did not significantly affect CM’s anti-tumor actions^15^. Third, in vivo experiments using a mouse model examined the effects of CM on lung invasion. The other frequent sites of PDAC metastasis include the abdomen, liver, bone, and brain, and these organs were not tested in this study^46^. Furthermore, the current study employed an immune-deficient mouse model and did not examine the influence of the immune microenvironment on the therapeutic potential of iTSCs. Future studies should continue to examine the dose–response effects of CM using primary PDAC cells and other in vivo primary and secondary tumor models. Of note, the efficacy of YS49 treatment differs depending on the type of host cells to generate iTSCs.

This study demonstrated that K-Ras and PI3K/PKA-activated MSC-derived CM as well as PBMC-derived CM have the capability of suppressing the proliferation and migration in several different pancreatic cancer cell lines. We envision that CM or proteins isolated from CM can be a new potential treatment that works alone or in combination with current chemotherapy agents and can help lead to a new frontier in the battle against pancreatic cancer.

## Materials and methods

### Cell culture

Human pancreatic cancer cells such as PANC1 (ATCC, Manassas, VA, USA), ASPC1 (obtained from Dr. Lin at Indiana University Purdue University Indianapolis, Indianapolis, USA), PANC10.05, PANC198, and Pa03C (obtained from Dr. Fishel at Indiana University School of Medicine, Indianapolis, USA) were cultured in DMEM (#10-013-CV, Corning Inc., Corning, NY, USA)^47^. MDA-MB-231 breast cancer cells (ATCC) were also cultured in DMEM^48^. Human MSCs (#PT-2501, Lonza, Basel, Swiss) were grown in mesenchymal stem cell basal medium (#PT-3001, Lonza) and cultured on a collagen-coated culture dish^15^. Jurkat T lymphocytes (ATCC) were cultured in RPMI1640 (#10-040-CV, Corning). The culture media was supplemented with 10% fetal bovine serum (FBS) and 1% antibiotics, and cells were maintained at 37 °C and 5% CO_2_. Cells were treated with 50 µM of YS49 (#HY-15477)^16^, MedChemExpress, Monmouth Junction, NJ, USA) as an activator of PI3K signaling or 20 µM CW008 (#5495, Tocris Bioscience, Bristol, UK)^49^ as an activator of PKA signaling for 24 h. Tumor cells were treated with 1 µg/mL of recombinant MSN protein (#MBS2031729; MyBioSource, San Diego, CA, USA)^15^ or 1 µg/mL MSN plus 0.5 µM Gemcitabine (#3259, Tocris Bioscience)^50^ for 24 h.

For in vitro experiments, MSC CM was prepared from 4 × 10^6^ cells in a 20 mL DMEM culture medium with antibiotics and a fraction of FBS consisting of 3 kD or smaller proteins. After one day of incubation, we collected the medium directly or the medium was condensed 20-fold using a filter (#R9MA86732, Sigma, St. Louis, MO, USA) at 800 × g and centrifuged for 30 min to collect proteins at 3 kD or heavier. Tumor cell-derived CM (1 × 10^6^ cells) was collected and centrifuged at 180×*g* for 5 min to remove cell debris. MSC CM was applied to tumor cells after completely removing the original medium. For in vivo experiments, MSC CM was harvested from the FBS-free medium and treated by a filter (#0000215519, Sigma) with a cutoff molecular weight of 3 kD.

### Human peripheral blood mononuclear cells (PBMCs)

Human peripheral blood samples were used based on the guidelines of the Declaration of Helsinki. The use of blood samples was approved by the Ethics Committee of Osaka University (protocol #21344). Informed consent was obtained from all subjects. Eight mL of peripheral blood was collected in a vacuum blood collection tube (VP-NA070K, Terumo, Tokyo, Japan) from 10 healthy volunteers (mean of 34.2 years, ranging from 23 to 54 years). An equal volume of 0.9% NaCl solution was mixed with blood samples, and 6 mL of the mixed solution was added with 3 mL of a lymphoprep solution (#1114544, Abbott Diagnostics Technologies AS, Norway). The mix solution was centrifuged at 800×*g* for 30 min at room temperature. Using a Pasteur pipette, PBMCs were carefully collected, and they were cultured in AlyS705 medium (Cell Science & Technology Institute, Inc., Sendai, Japan).

### MTT assay

Approximately 2000 cells were seeded on 96-well plates (#3585, Corning). The treatment agents such as CM, drugs, or proteins were added the next day, and cells were incubated at 37 °C for two days. Cells were then stained with 0.5 mg/mL thiazolyl blue tetrazolium bromide (#M5655, Sigma) for 4 h. A mixed solution of isopropyl alcohol (#A416-4, Thermo-Fisher) and hydrochloric acid (#3750-32, Ricca Chemical, Arlington, TX, USA) was added at the ratio of 60:1. Optical density for assessing metabolic activities was determined at 570 nm. The relative cell viability was determined as an absorbance ratio of each sample to a control.

### EdU assay

Approximately 1000 cells were seeded on 96-well plates (Corning) on day 1, and the treatment agents such as ​CM, drugs, or proteins were added on day 2. Cells were cultured for two days and incubated with 10 µM EdU (Click-iT™ EdU Alexa Fluor™ 488 Imaging Kit; Thermo-Fisher)^51^ for 4 h at dark. Cells were fixed in a 3.7% formaldehyde solution for 15 min and washed with PBS. Cells were incubated with a Click-iT reaction cocktail (Thermo-Fisher) in the dark for 30 min. Green fluorescent images were taken and the number of fluorescently labeled cells was counted using Image J software (NIH, Bethesda, MD, USA).

### Transwell invasion assay

The invasion ability of cancer cells was determined using 12-well plates (#35-3043, Corning) and transwell chambers with an 8 µm transparent membrane (#353182, Corning). We prepared 300 µL Matrigel (100 µg/mL) in the transwell chamber on day 1. Approximately 0.5 × 10^5^ cells were seeded in 300 µL of serum-free DMEM and placed in the upper layer of the transwell, while 800 µL of DMEM was placed in the lower layer. MSC CM was then applied to the upper layer of the transwell. After 2-day incubation, cells were stained with Crystal Violet (diluted 1:25 in water) for 30 min, and the number of invaded cells was counted.

### Scratch assay

A wound-healing scratch assay was utilized to evaluate the two-dimensional migratory behavior of cancer cells by measuring the scratch area^52^. Approximately 2 × 10^5^ cells were seeded in 12-well plates on day 1. On day 2, a scratch was made on the cell layer with a tip of a plastic pipette. Cells were washed with DMEM to remove floating cells and MSC CM was applied. Images of cell-free zones were taken at 0 h and 24 h with a light microscope (40 × magnification), and the areas of the cell-free zone were quantified by Image J.

### Western blot analysis

Cells were lysed with a RIPA lysis buffer (#sc-24948, Santa Cruz Biotechnology, Dallas, TX, USA) with protease inhibitors (#PIA32963, Thermo-Fisher), phosphatase inhibitors (#2006643, Calbiochem, Billerica, MA, USA). Proteins were separated by 10–15% SDS gel (Bio-Rad Laboratories, Hercules, CA, USA) and transferred to a polyvinylidene difluoride membrane (#IPVH00010, Sigma). The membrane was incubated overnight with the primary antibodies, followed by incubation with secondary antibodies for 45 min (7074S/7076S, Cell Signaling, Danvers, MA, USA). We used antibodies against cMyc, p-Akt, Akt, Snail, MSN, CD44, p-CREB, CREB (Cell Signaling), and K-Ras (Santa Cruz Biotechnology), with β-actin (Sigma) as a control.

### Plasmid and siRNA transfection

Plasmids for cMyc and K-Ras (#17758 and #159554, respectively, Addgene, Watertown, MA, USA) were transfected to overexpress cMyc and K-Ras, respectively, while a blank plasmid vector (FLAG-HA-pcDNA3.1; Addgene) was used as a control. Plasmids were transfected to 70–90% confluent cells using lipofectamine^®^3000 (#L300015, Thermo-Fisher). RNA interference was conducted to silence CD44 using its specific siRNA (#s63659, Thermo-Fisher), with a nonspecific control siRNA (Silencer Select #1, Life Technologies, CO, USA). Transfection was conducted using lipofectamine RNAiMAX (#13778075, Life Technologies), and the efficiency of silencing was assessed with Western blotting 24 h after transfection.

### Immunoprecipitation

Protein samples were pretreated with agarose beads (#GE17-6002-35, Sigma) conjugated with protein A and rabbit IgG, followed by overnight immunoprecipitation with the beads conjugated with anti-MSN antibodies (Cell Signaling). The beads were collected by centrifugation, washed three times with PBS, and resuspended for Western blotting. Western blotting was conducted using antibodies against CD44 (Cell Signaling).

### Ex vivo tissue assay

The usage of human pancreatic cancer was approved by the Indiana University Institutional Review Board (#1911155674), and the tissues were received from Indiana University Simon Comprehensive Cancer Center Tissue Procurement Core. Informed consent was obtained from the subject. A freshly isolated pancreatic cancer tissue (~ 1 g) was manually fragmented with a scalpel into small pieces (0.5–0.8 mm in length), which were grown in DMEM with 10% FBS and antibiotics for a day. The described CM was then added for 4 additional days, and a change in the fragment size was determined.

### Animal model and histology

The experimental procedures using animals were approved by the Indiana University Animal Care and Use Committee (#SC330R) and complied with the Guiding Principles in the Care and Use of Animals endorsed by the American Physiological Society. The animal experiments were reported following ARRIVE guidelines^53^. Mice were housed in five per cage and provided with mouse chow and water ad libitum. To evaluate the effects of CMs on tumor invasion, an in vivo extravasation assay was conducted. Pa03C tumor cells (~ 1.0 × 10^5^ cells in 100 µL PBS), which were labeled with red fluorescence, were inoculated to NSG female mice (~ 8 weeks old) from the tail vein. Animals were randomized using a stratified randomization procedure by considering body weight, and three groups such as placebo (injection of control CM), MSC CM, and YS49-treated MSC CM were employed (5 mice per group). The placebo mice received a daily i.v. injection of the control medium from the tail vein, while mice in the other two groups received MSC CM or YS49-treated MSC CM. Mice were sacrificed in 2 weeks by the inhalation of CO_2_, and the presence of tumor cells in the lung was determined histologically. H&E staining was conducted as described previously^54^. No animals or data points were excluded.

### Statistical analysis

For cell-based experiments, three or four independent experiments were conducted, and data were expressed as mean ± SD. In animal experiments, the sample size in the mouse model was chosen to achieve a power of 80% with p < 0.05. Statistical significance was evaluated using a one-way analysis of variance (ANOVA) and Student’s t-test (MS Excel). The single and double asterisks in the figures indicate p < 0.05 and p < 0.01, respectively, and statistical significance was evaluated at p < 0.05. In the survival analysis of TCGA, the high and low expression levels were determined based on the 50% (medium) cutoff.

### Ethical approval

The experimental procedures using animals were approved by the Indiana University Animal Care and Use Committee (#SC330R) and complied with the Guiding Principles in the Care and Use of Animals endorsed by the American Physiological Society.

### Supplementary Information

Supplementary Figures.

## Data Availability

All data relevant to the study are included in the article or uploaded as supplementary information.

## References

[1] Heller DR, Nicolson NG, Ahuja N, Khan S, Kunstman JW (2020). Association of treatment inequity and ancestry with pancreatic ductal adenocarcinoma survival. JAMA Surg..

[2] Fulop DJ, Zylberberg HM, Wu YL (2023). Association of antibiotic receipt with survival among patients with metastatic pancreatic ductal adenocarcinoma receiving chemotherapy. JAMA Netw. Open..

[3] Grossberg AJ, Chu LC, Deig CR (2020). Multidisciplinary standards of care and recent progress in pancreatic ductal adenocarcinoma. CA Cancer J. Clin..

[4] Otsuka H, Uemura K, Kondo N (2020). Clinical characteristics of initial recurrence in lung after surgical resection for pancreatic ductal adenocarcinoma. Pancreatology.

[5] Torphy RJ, Fujiwara Y, Schulick RD (2020). Pancreatic cancer treatment: better, but a long way to go. Surg Today..

[6] DiMagno EP, Reber HA, Tempero MA (1999). AGA technical review on the epidemiology, diagnosis, and treatment of pancreatic ductal adenocarcinoma. Am. Gastroenterol. Assoc. Gastroenterol..

[7] Keleg S, Buchler P, Ludwig R, Buchler MW, Friess H (2003). Invasion and metastasis in pancreatic cancer. Mol. Cancer..

[8] Conroy T, Desseigne F, Ychou M (2011). FOLFIRINOX versus gemcitabine for metastatic pancreatic cancer. N. Engl. J. Med..

[9] Von Hoff DD, Ervin T, Arena FP (2013). Increased survival in pancreatic cancer with nab-paclitaxel plus gemcitabine. N. Engl. J. Med..

[10] Moore MJ, Goldstein D, Hamm J (2007). Erlotinib plus gemcitabine compared with gemcitabine alone in patients with advanced pancreatic cancer: A phase III trial of the National Cancer Institute of Canada Clinical Trials Group. J. Clin. Oncol..

[11] Burris HA, Moore MJ, Andersen J (1997). Improvements in survival and clinical benefit with gemcitabine as first-line therapy for patients with advanced pancreas cancer: A randomized trial. J. Clin. Oncol..

[12] Li KX, Sun X, Li BY, Yokota H (2021). Conversion of osteoclasts into bone-protective, tumor-suppressing cells. Cancers (Basel)..

[13] Sun X, Li K, Li B-Y, Yokota H (2022). Wnt signaling: A double-edged sword in protecting bone from cancer. J. Bone Miner. Metab..

[14] Liu S, Wu D, Sun X (2021). Overexpression of Lrp5 enhanced the anti-breast cancer effects of osteocytes in bone. Bone Res..

[15] Sun X, Li K, Hase M (2022). Suppression of breast cancer-associated bone loss with osteoblast proteomes via Hsp90ab1/moesin-mediated inhibition of TGFbeta/FN1/CD44 signaling. Theranostics..

[16] Sun X, Li K, Aryal UK, Li BY, Yokota H (2022). PI3K-activated MSC proteomes inhibit mammary tumors via Hsp90ab1 and Myh9. Mol. Ther. Oncolytics..

[17] Li K, Sun X, Zha R (2022). Counterintuitive production of tumor-suppressive secretomes from Oct4- and c-Myc-overexpressing tumor cells and MSCs. Theranostics..

[18] Liu S, Sun X, Li K (2021). Generation of the tumor-suppressive secretome from tumor cells. Theranostics..

[19] Sun X, Li K, Zha R (2021). Preventing tumor progression to the bone by induced tumor-suppressing MSCs. Theranostics..

[20] Sano T, Sun X, Feng Y (2021). Inhibition of the growth of breast cancer-associated brain tumors by the osteocyte-derived conditioned medium. Cancers (Basel)..

[21] Takeuchi R, Katagiri W, Endo S, Kobayashi T (2019). Exosomes from conditioned media of bone marrow-derived mesenchymal stem cells promote bone regeneration by enhancing angiogenesis. PLoS ONE.

[22] Li K, Sun X, Minami K (2023). Proteomes from AMPK-inhibited peripheral blood mononuclear cells suppress the progression of breast cancer and bone metastasis. Theranostics..

[23] Kanda M, Matthaei H, Wu J (2012). Presence of somatic mutations in most early-stage pancreatic intraepithelial neoplasia. Gastroenterology.

[24] Jones S, Zhang X, Parsons DW (2008). Core signaling pathways in human pancreatic cancers revealed by global genomic analyses. Science.

[25] Strickler JH, Satake H, George TJ (2023). Sotorasib in KRAS p.G12C-mutated advanced pancreatic cancer. N. Engl. J. Med..

[26] Indini A, Rijavec E, Ghidini M, Cortellini A, Grossi F (2021). Targeting KRAS in solid tumors: Current challenges and future opportunities of novel KRAS inhibitors. Pharmaceutics..

[27] Adderley H, Blackhall FH, Lindsay CR (2019). KRAS-mutant non-small cell lung cancer: Converging small molecules and immune checkpoint inhibition. EBioMedicine.

[28] Thein KZ, Biter AB, Hong DS (2021). Therapeutics targeting mutant KRAS. Annu. Rev. Med..

[29] McCubrey JA, Steelman LS, Abrams SL (2006). Roles of the RAF/MEK/ERK and PI3K/PTEN/AKT pathways in malignant transformation and drug resistance. Adv. Enzyme Regul..

[30] Kwan AK, Piazza GA, Keeton AB, Leite CA (2022). The path to the clinic: A comprehensive review on direct KRAS(G12C) inhibitors. J. Exp. Clin. Cancer Res..

[31] Skoulidis F, Li BT, Dy GK (2021). Sotorasib for lung cancers with KRAS p.G12C mutation. N. Engl. J. Med..

[32] de la Cova C, Abril M, Bellosta P, Gallant P, Johnston LA (2004). Drosophila myc regulates organ size by inducing cell competition. Cell.

[33] Senoo-Matsuda N, Johnston LA (2007). Soluble factors mediate competitive and cooperative interactions between cells expressing different levels of Drosophila Myc. Proc. Natl. Acad. Sci. U. S. A..

[34] Ahmed MB, Alghamdi AAA, Islam SU, Lee JS, Lee YS (2022). cAMP signaling in cancer: A PKA-CREB and EPAC-centric approach. Cells.

[35] Zhang J-G, Zhao G, Qin Q (2013). Nicotinamide prohibits proliferation and enhances chemosensitivity of pancreatic cancer cells through deregulating SIRT1 and Ras/Akt pathways. Pancreatology.

[36] Sun X, Li KX, Figueiredo ML, Lin CC, Li BY, Yokota H (2022). Generation of the chondroprotective proteomes by activating PI3K and TNFalpha signaling. Cancers (Basel)..

[37] Liu, X. *The Role of Mir-218 in Breast Cancer Bone Metastasis* (City of Hope's Irell & Manella Graduate School of Biomedical Sciences, 2018).

[38] Li K, Sun X, Li H (2022). Suppression of osteosarcoma progression by engineered lymphocyte-derived proteomes. Genes Dis..

[39] Zhao S, Chen C, Chang K (2016). CD44 expression level and isoform contributes to pancreatic cancer cell plasticity, invasiveness, and response to therapy. Clin. Cancer Res..

[40] Gao F, Zhang G, Liu Y (2022). Activation of CD44 signaling in leader cells induced by tumor-associated macrophages drives collective detachment in luminal breast carcinomas. Cell Death Dis..

[41] Li XP, Zhang XW, Zheng LZ, Guo WJ (2015). Expression of CD44 in pancreatic cancer and its significance. Int. J. Clin. Exp. Pathol..

[42] Adhikari H, Kattan WE, Kumar S, Zhou P, Hancock JF, Counter CM (2021). Oncogenic KRAS is dependent upon an EFR3A-PI4KA signaling axis for potent tumorigenic activity. Nat. Commun..

[43] Baleeiro RB, Bouwens CJ, Liu P (2022). MHC class II molecules on pancreatic cancer cells indicate a potential for neo-antigen-based immunotherapy. Oncoimmunology..

[44] Leidner R, Sanjuan Silva N, Huang H (2022). Neoantigen T-cell receptor gene therapy in pancreatic cancer. N. Engl. J. Med..

[45] Blair HA (2021). Sotorasib: First approval. Drugs.

[46] Liu Q, Zhang R, Michalski CW, Liu B, Liao Q, Kleeff J (2020). Surgery for synchronous and metachronous single-organ metastasis of pancreatic cancer: A SEER database analysis and systematic literature review. Sci. Rep..

[47] Winnicka K, Bielawski K, Bielawska A, Sura SA (2008). Antiproliferative activity of derivatives of ouabain, digoxin and proscillaridin A in human MCF-7 and MDA-MB-231 breast cancer cells. Biol. Pharm. Bull..

[48] Lee T-H, Avraham HK, Jiang S, Avraham S (2003). Vascular endothelial growth factor modulates the transendothelial migration of MDA-MB-231 breast cancer cells through regulation of brain microvascular endothelial cell permeability*. J. Biol. Chem..

[49] Li K, Huo Q, Dimmitt NH (2023). Osteosarcoma-enriched transcripts paradoxically generate osteosarcoma-suppressing extracellular proteins. Elife.

[50] Amrutkar M, Vethe NT, Verbeke CS (2020). Differential gemcitabine sensitivity in primary human pancreatic cancer cells and paired stellate cells is driven by heterogenous drug uptake and processing. Cancers (Basel)..

[51] Liu S, Fan Y, Chen A (2018). Osteocyte-driven downregulation of snail restrains effects of Drd2 inhibitors on mammary tumor cells. Can. Res..

[52] Liu S, Liu Y, Minami K (2018). Inhibiting checkpoint kinase 1 protects bone from bone resorption by mammary tumor in a mouse model. Oncotarget.

[53] Percie du Sert N, Hurst V, Ahluwalia A (2020). The ARRIVE guidelines 2.0: Updated guidelines for reporting animal research. PLoS Biol..

[54] Xu W, Wan Q, Na S, Yokota H, Yan J-L, Hamamura K (2015). Suppressed invasive and migratory behaviors of SW1353 chondrosarcoma cells through the regulation of Src, Rac1 GTPase, and MMP13. Cell. Signal..

