# Supplementary material for: The inhibition of pancreatic cancer progression by K-Ras-overexpressing mesenchymal stem cell-derived secretomes
Source: Sci Rep. 2023 Sep 12;13:15036. doi: 10.1038/s41598-023-41835-6 (PMC10497626; doi:10.1038/s41598-023-41835-6)
Supplement: Supplementary file 1 — Supplementary Figures. [file 41598_2023_41835_MOESM1_ESM.pdf]

Supplementary Information

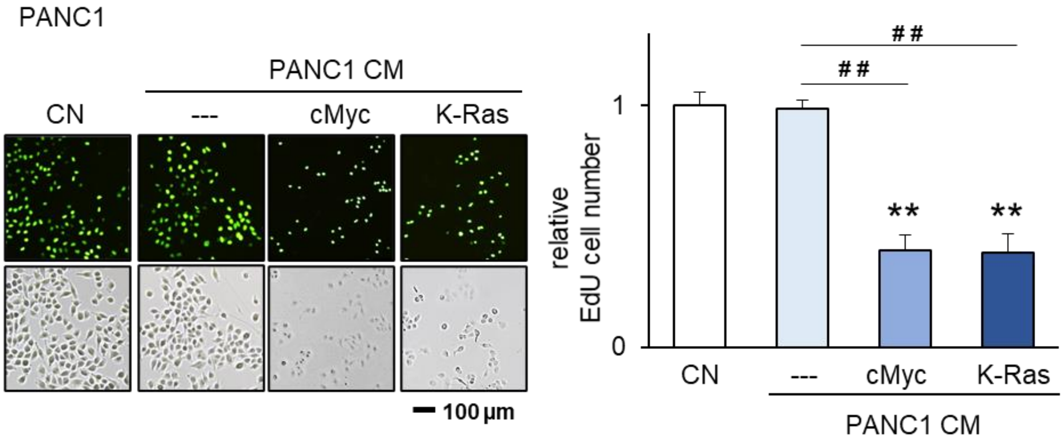

Suppl. Figure 1. Tumor-suppressive CM, derived from PANC1 pancreatic cancer cells. The double asterisk (comparison to CN) and pound sign (comparison to CM control) indicate 0.01. CM = conditioned medium, CN = control. Reduction in EdU-based proliferation by cMyc- and K-Ras-overexpressing PANC1-derived CM.

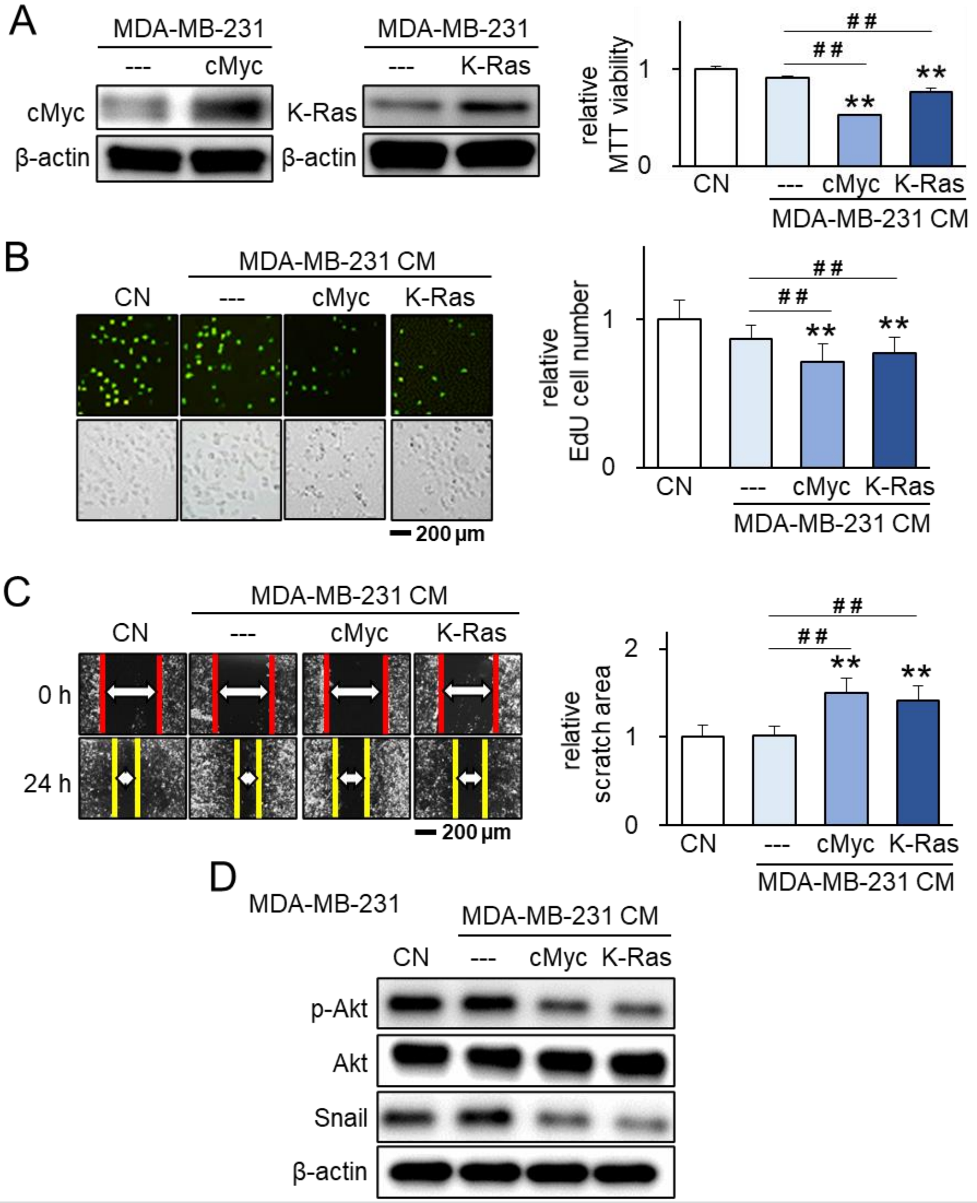

Suppl. Figure 2. Tumor-suppressive CM, derived from MDA-MB-231 breast cancer cells. The double asterisk (comparison to CN) and pound sign (comparison to CM control) indicate 0.01. CM = conditioned medium, CN = control. (A-C) Generation of tumor-suppressive CM from MDA-MB-231 breast cancer cells by overexpressing cMyc and K-Ras. The MTT-based viability, EdU-based proliferation, and scratch-based motility of MDAN-MB-231 cells were reduced by the tumor-suppressive CM, respectively. (D) Downregulation of p-Akt and Snail by cMyc- and K-Ras-overexpressing MDA-MB-231 cell-derived CM.

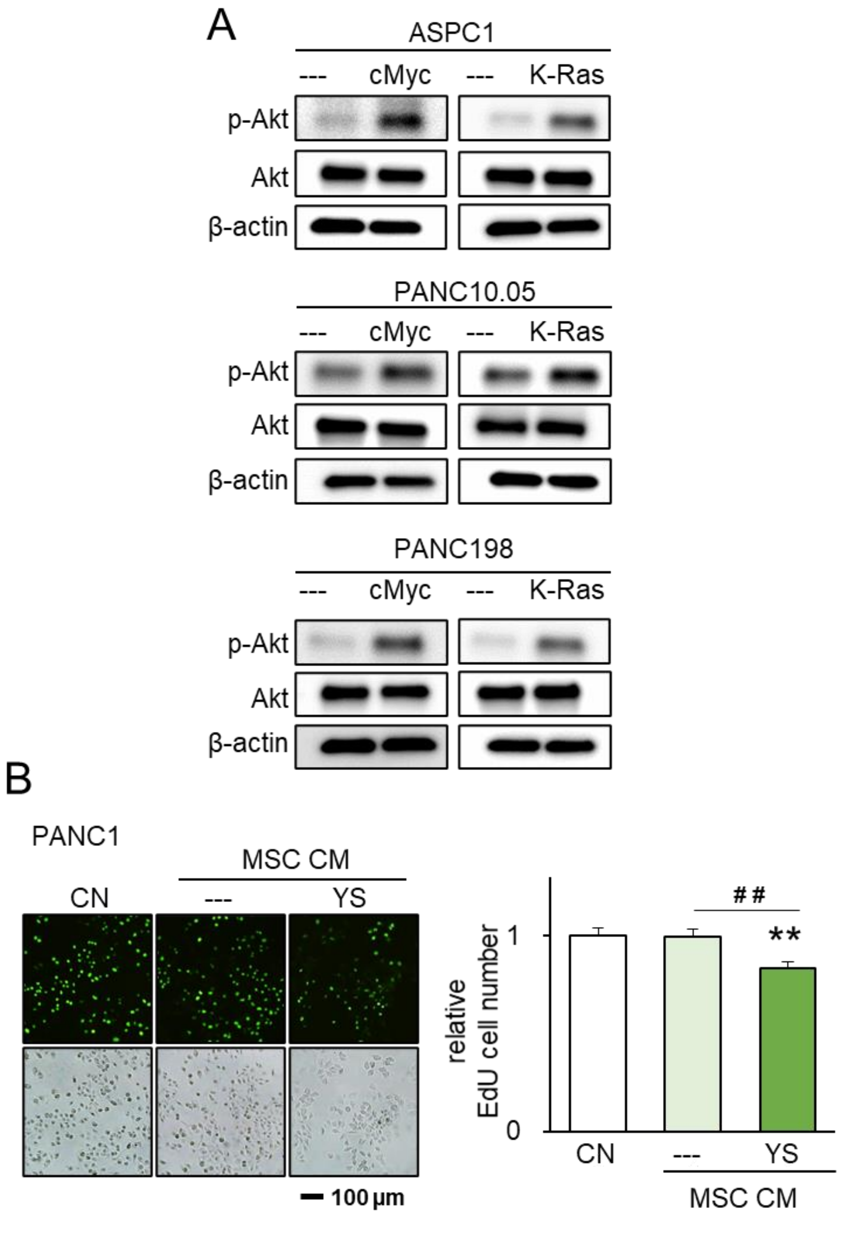

Suppl. Figure 3. **Elevation of p-Akt in the three PANC cell lines in response to the overexpression of cMyc and K-Ras, and tumor-suppressive capability of PI3K-activated MSC-derived CM with PANC1 pancreatic cancer cells.** The double asterisk (comparison to CN) and pound sign (comparison to CM control) indicate 0.01. CN = control, CM = conditioned media, and YS = YS49, PI3K activator. (A) Elevation in p-Akt by cMyc and K-Ras overexpression in ASPC1, PANC10.05 and PANC198 cells. (B) Reduction in EdU-based proliferation by YS49-treated MSC-derived CM.

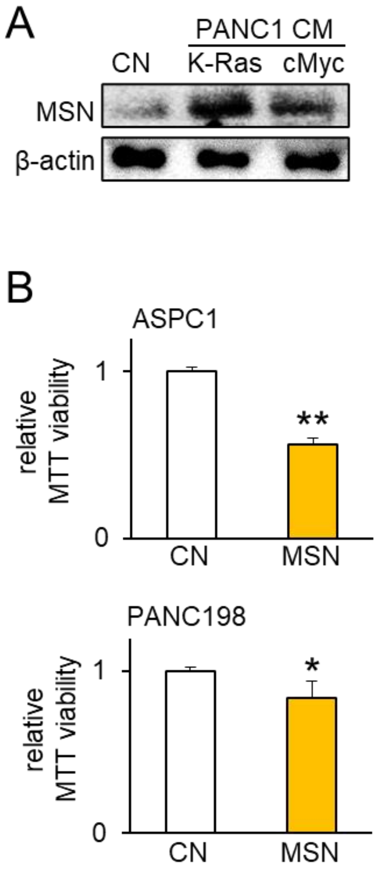

Suppl. Figure 4. **Elevation of MSN in K-Ras and cMyc-overexpressing PANC1-derived CM, and tumor-suppressive capability of MSN recombinant protein.** The single and double asterisks indicate $p < 0.05$ and 0.01, respectively. CN = control. (A) Elevated level of MSN in PANC1 cells by K-Ras- or cMyc-overexpressing PANC1-derived CM. (B) Reduction in the MTT-based viability of ASPC1 and PANC198 pancreatic cancer cells in response to 1 μg/mL of recombinant MSN proteins.

Uncropped Gels

Figure 1

Fig. 1E left

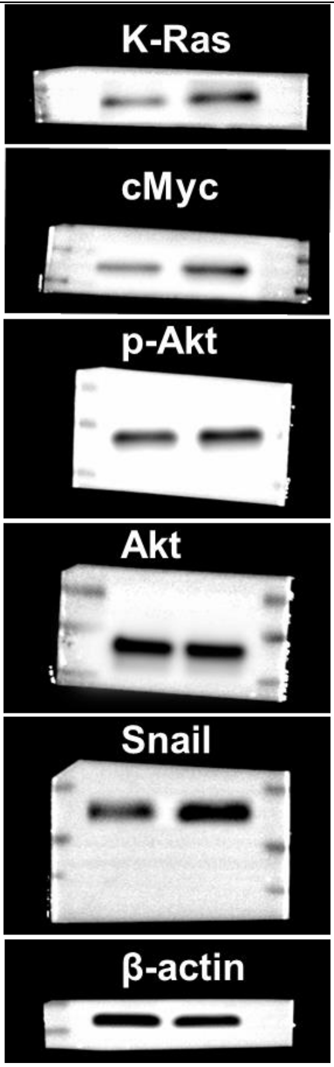

Fig. 1E right

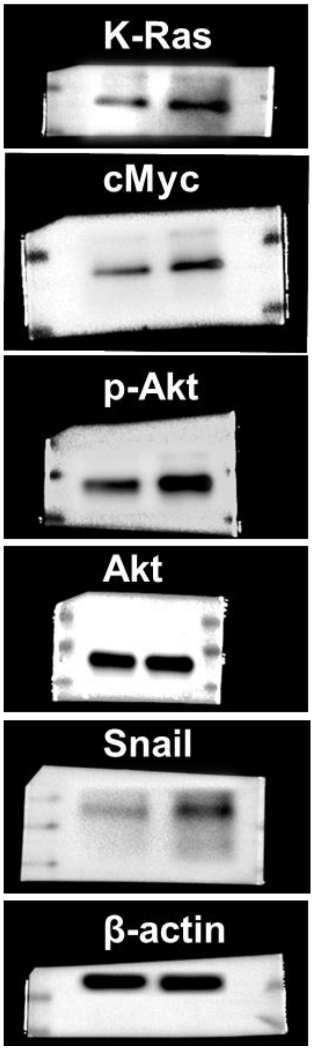

Fig. 1G

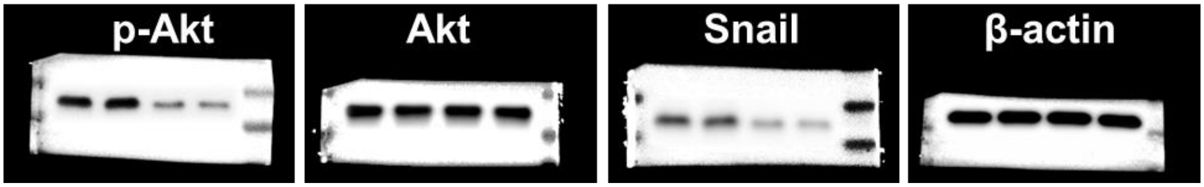

Figure 2

Fig. 2A

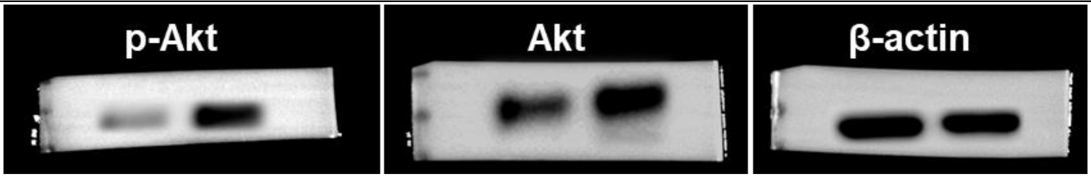

Fig. 2E left

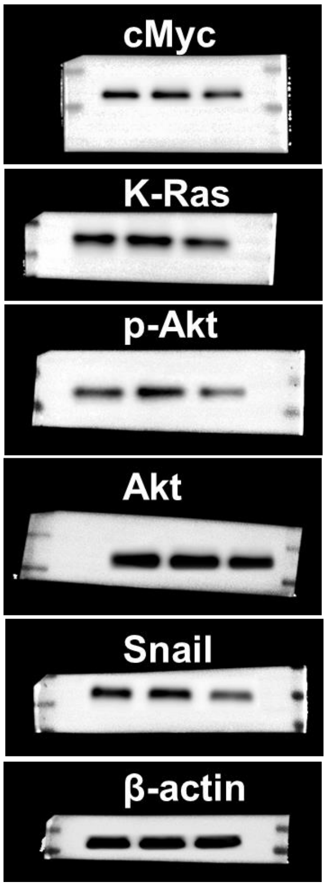

Fig. 2E right

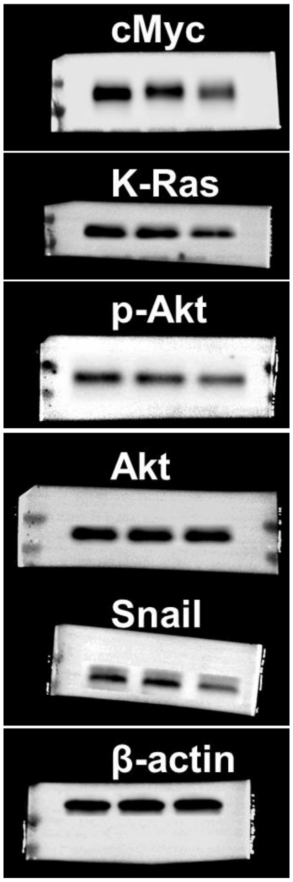

Fig. 2F

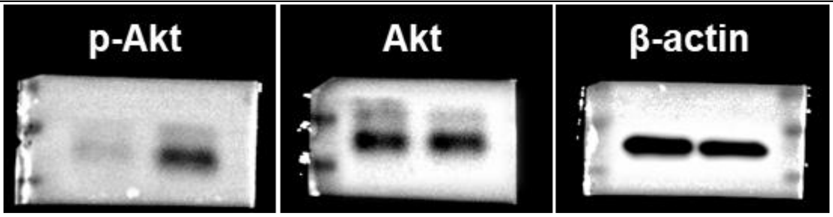

Figure 4

Fig. 4A

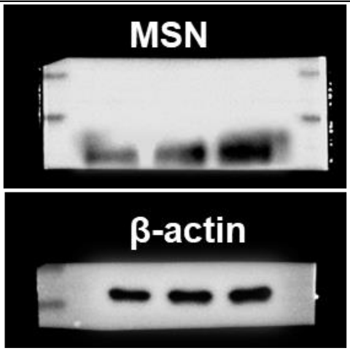

Fig. 4D

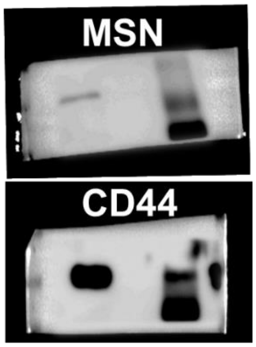

Fig. 4E up

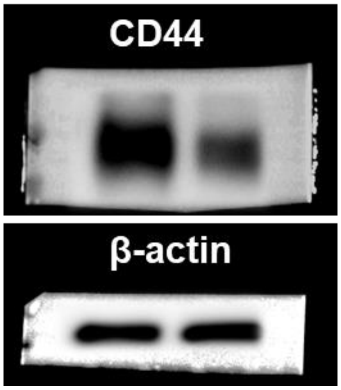

Fig. 4E down

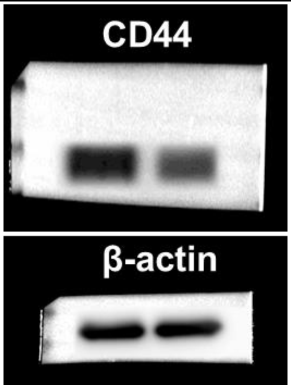

Fig. 4G

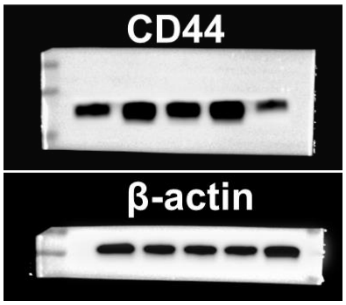

Fig. 4H up

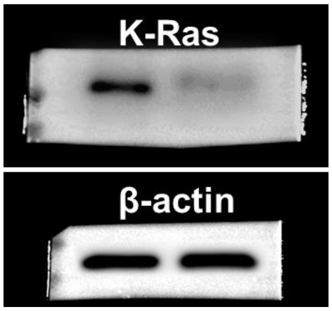

Fig. 4H down

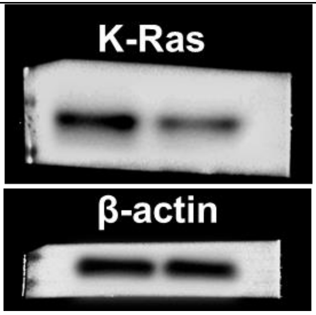

Figure 6

Fig. 6C

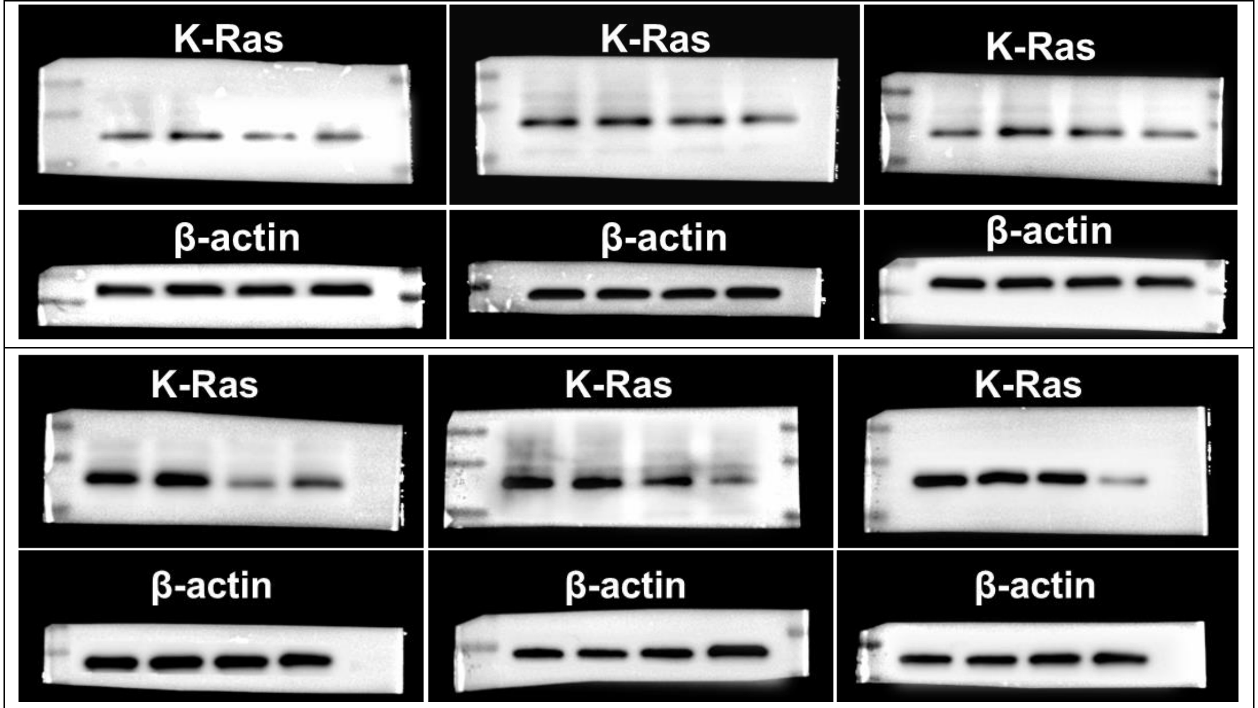

Figure 7

Fig. 7B left	Fig. 7B right
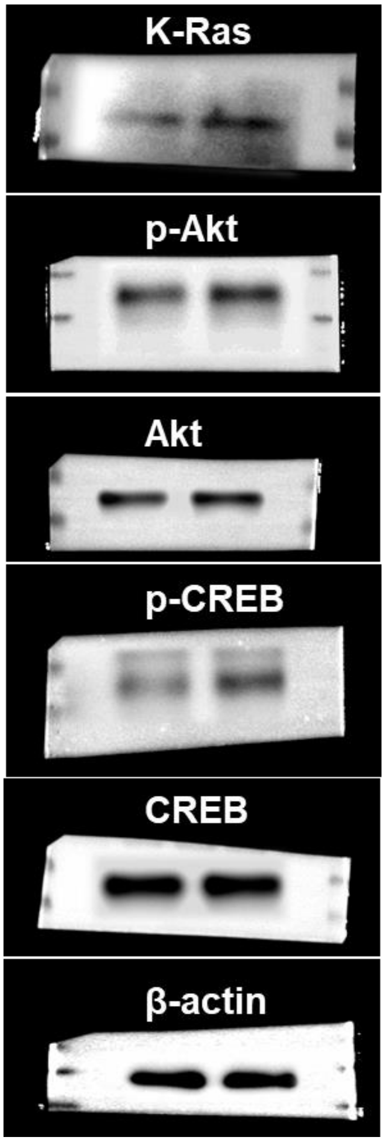	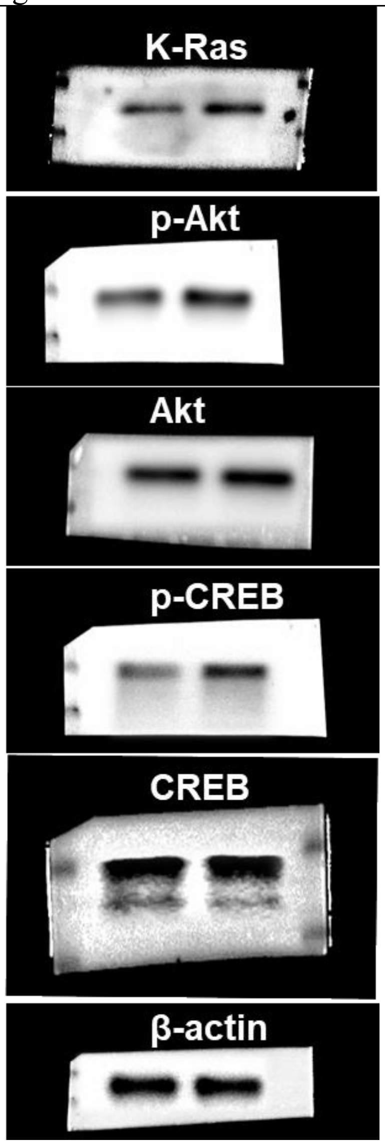

Suppl. Fig. 2

<table>
<tr><td>Suppl. Fig. 2A left</td><td>Suppl. Fig. 2A right</td></tr>
<tr><td>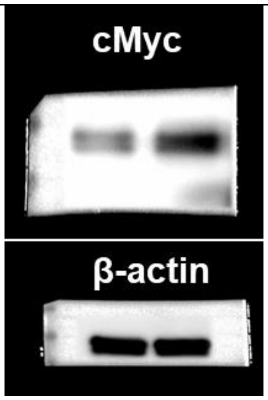
</td><td>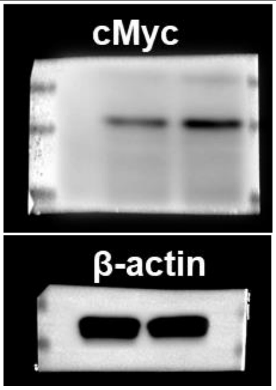
</td></tr>
<tr><td colspan="2">Suppl. Fig. 2D</td></tr>
<tr><td colspan="2">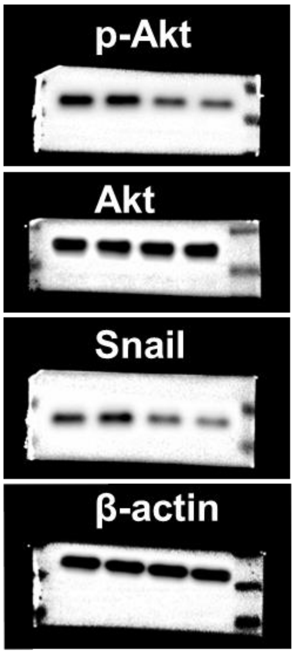
</td></tr>
</table>

Suppl. Fig. 3

Suppl. Fig. 3A

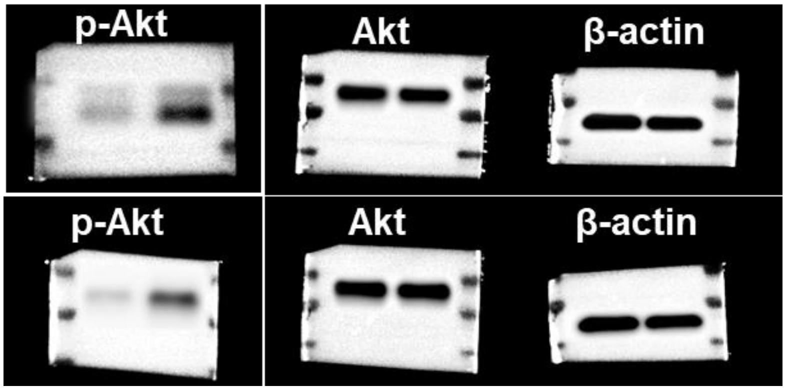

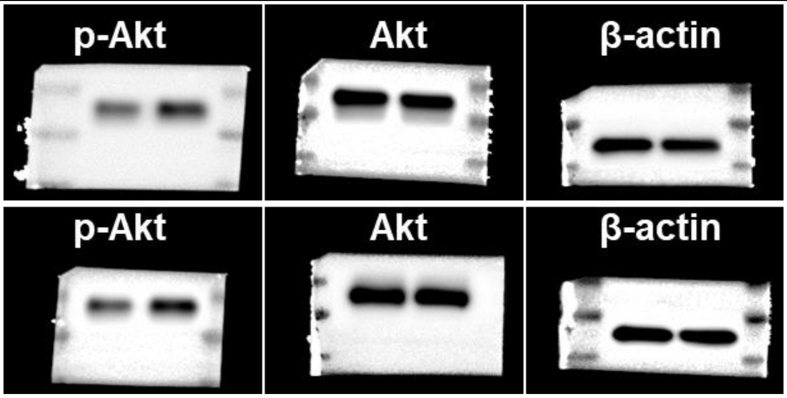

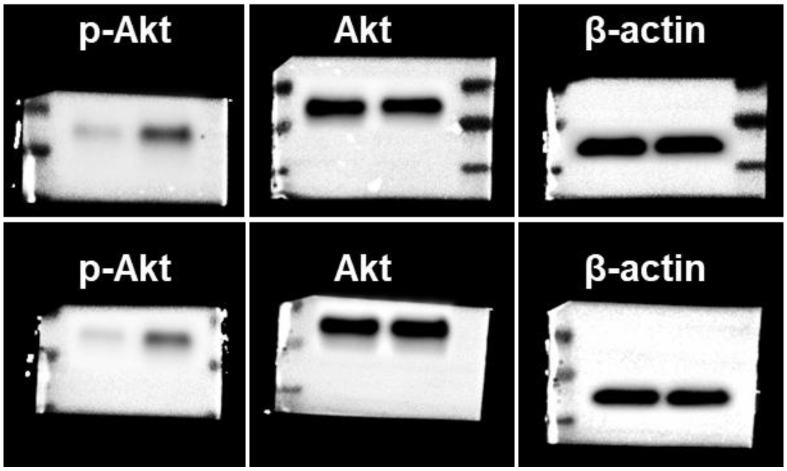

Suppl. Fig. 4

Suppl. Fig. 4A
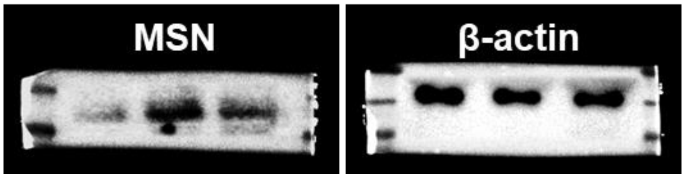
